# A CLEIA Antigen Assay in Diagnosis and Follow-Up of SARS-CoV-2-Positive Subjects

**DOI:** 10.1128/spectrum.01032-21

**Published:** 2022-05-02

**Authors:** Salvatore Scarcella, Azzurra Rizzelli, Andrea Fontana, Chiara Zecca, Giancarlo Pasanisi, Katia Musio, Anna Laura Putignano, Valerio Aprile, Alberto Fedele, Pierangelo Errico, Massimiliano Copetti, Vittorio Tassi

**Affiliations:** a Laboratory Medicine Unit, Ospedale Card. G. Panico, Tricase, Italy; b Unit of Biostatistics, Fondazione IRCCS Casa Sollievo della Sofferenza, San Giovanni Rotondo, Italy; c Prevention Department, Hygiene and Public Health Service, Local Health Unit, Lecce, Italy; d Health Management Unit, Ospedale Card. Panico, Tricase, Lecca, Italy; Keck School of Medicine, University of Southern California

**Keywords:** SARS-CoV-2, nasopharyngeal swabs, antigenic test

## Abstract

This study includes 259 consecutive nasopharyngeal swabs which tested positive for a molecular SARS-CoV-2 test and 77 subjects who were followed longitudinally, with nasopharyngeal swabs performed weekly until clinical recovery and a negative result for the molecular test were reached. All swabs were also tested with a Lumipulse SARS-CoV-2 chemiluminescence enzyme immunoassay (CLEIA) antigen assay. The antigen test was positive in 169 (65.3%) out of the 259 subjects, while no antigen was detected in 90 subjects (34.7%). In the antigen-positive subjects, clinical status moved slightly toward a more frequent presence of symptoms. Longitudinal follow-up shows how the time of negativization has a faster kinetic in the antigenic test than in the molecular test. Antigenic test result values, considered as a time-dependent covariate and log-transformed, were highly associated with the time to negative swab, with good prediction ability. Receiver operating characteristic (ROC) curve analysis showed a very good discrimination ability of antigenic tests in classifying negative swabs. The optimal cutoff which jointly maximized sensitivity and specificity was 1.55, resulting in an overall accuracy of 0.75, a sensitivity of 0.73, and a specificity of 0.83. After dichotomizing the antigenic test according to the previously determined cutoff value of 1.55, the time-dependent covariate Cox model again suggests a highly significant association of antigenic test values with the time to negative swab molecular: a subject with an antigenic test value lower than 1.55 had almost a 13-fold higher probability to also result negative in the molecular test compared to a subject with an antigenic test value higher than 1.55.

**IMPORTANCE** Our work explores the possibility of using a sensible and reliable antigenic test in a wider range of SARS-CoV-2 diagnostic and clinical applications. Furthermore, this tool seems particularly promising in follow-up with infected subjects, because while the molecular test frequently yields the persistence of low positivities, raising yet unanswered questions, this antigenic test shows more uniform and faster negativization during the evolution of the infection, somehow paralleling the dynamics of infectivity. Although more data will be required to definitely prove it, we believe these findings might be of great interest.

## INTRODUCTION

The COVID-19 pandemic is an unprecedented global health crisis, impacting 192 out of 200 countries in the world, with 247,719,391 people infected and a total of 5,016,369 deaths in approximately 21 months as of 2 November 2021 (1). Due to this pandemic, most of the world’s population have profoundly modified their normal activity, mostly confining themselves at home, which has severely affected the global economy. The pathogenic agent of COVID-19 is a highly contagious respiratory RNA virus, SARS-CoV-2, a new strain of β-coronavirus. Early diagnosis represents a fundamental tool to identify infected people as soon as possible, helping to cut the spread of the disease. Furthermore, by tracing subjects who are in contact with infected people, prompt diagnosis allows sanitization of the affected area, thus contributing to reducing the wide spread of the disease.

Testing for SARS-CoV-2 on respiratory samples, mainly rhinopharyngeal swabs, has predominantly been performed utilizing molecular platforms, with real-time PCR (RT-PCR) being considered the gold standard of techniques so far. Its analytical sensitivity and specificity are very high, allowing the detection of even a few viral RNA copies.

Such high sensitivity is a double-edged sword, however, since although it is possible to detect very low viral loads, the possibility of false positives (2) cannot be ruled out. Furthermore, weak positivity is often detected in patients for weeks to months following initial infection (3–4), and it is not still clear weather the widely used molecular methodology detects a low number of complete RNA copies of the virus, or chunks of it, which may not necessarily correlate with an active infection. (5–6). Therefore, since RT-PCR does not distinguish between infectious and noninfectious virus, propagating virus from clinical samples on cultured cells might be used as a proxy for infectiousness. Data from such an approach seems to confirm a decline in infectivity beyond 8 to 10 days from the onset of illness or the first positive RT-PCR result in asymptomatic subjects (7–8).

Since an assay on cultured cells is not easily available for diagnostic purposes, a more accessible test would be highly useful to pinpoint the moment at which infectivity declines to a negligible level, despite the persistent, though weak, positivity of the molecular test.

Recently, the Lumipulse SARS-CoV-2 antigen assay (Fujirebio, Inc., Tokyo, Japan), based on chemiluminescence enzyme immunoassay (CLEIA) technology and able to detect and quantitatively estimate the presence of SARS-CoV-2 nucleocapsid protein (NP) in nasopharyngeal swabs or saliva, was introduced in diagnostics. This test has optimal correlation with RT-PCR in the early phase of infection, which is characterized by high viral load, and does not seem to be affected by significant variability in the late phase of the infection when the RT-PCR signal may be varying (9–10).

The present work explores the possibility of using the Lumipulse SARS-CoV-2 antigen assay in a wider range of diagnostics and clinical applications, including follow-up with infected subjects.

## RESULTS

The present study includes 259 consecutive specimens with positive results for the molecular SARS-CoV-2 test, collected between 25 January and 18 February 2021. Out of the total, 242 were from the Public Hygiene and Prevention Service (SISP) of the Lecce province (Puglia region, Italy) and 17 were from Tricase Hospital (Pia Fondazione di Culto e Religione Cardinale Giovanni Panico). Of the total, 156 samples represented specimens collected following the initial diagnosis, while 103 represented newly diagnosed samples. Among the latter subjects, 77 were also followed longitudinally, with a nasopharyngeal swab performed weekly from 25 January to 11 March 2021, until clinical recovery and a negative result for the molecular test were reached.

The median age of participants was 51.45 years (range from 1.80 to 93.2 years), with 47% males and 53% females (Table 1). Of these, 178 (68.7%) were completely asymptomatic while 77 (29.7%) were mildly symptomatic; 4 (1.5%) had no clinical information.

**TABLE 1:** Baseline clinical and demographic characteristics of subjects with positive molecular test results (*n* = 259)^*a*^

Characteristic	Whole group (*n* = 259)	Ag-positive (*n* = 169)	Ag-negative (*n* = 90)	*P* (Ag-positive vs Ag-negative)
Age				
Mean (SD)	51.93 (22.68)	52.06 (21.73)	51.07 (29.49)	0.884
Median	51.45	51.21	55.01	
Range	1.80–93.22	2.46–93.22	1.80–85.40	
				
Sex, *n* (%)				
Female	137 (52.9)	91 (53.8)	46 (51.1)	0.675
Male	122 (47.1)	78 (46.2)	44 (48.9)	
				
Clinical status, *n* (%)				
Paucisymptomatic	77 (29.7)	59 (34.9)	18 (20.0)	0.040
Asymptomatic	178 (68.7)	108 (63.9)	70 (77.8)	
Unknown	4 (1.5)	2 (1.2)	2 (2.2)	
				
*C_T_* value molecular test				
Target “O”				
Mean (SD)		28.12 (6.52)	36.08 (2.16)	<0.001
Median (Q1, Q3)		29.00 (22.00, 33.00)	36.00 (35.00, 38.00)	
Range		16.00–40.00	29.00–42.00	
Non-missing		169	89	
Missing		0	1	
Target “R”				
Mean (SD)		29.54 (6.14)	36.50 (1.96)	<0.001
Median (Q1, Q3)		30.00 (24.00, 35.00)	37.00 (36.00, 38.00)	
Range		17.00–40.00	30.00–42.00	
Non-missing		168	80	
Missing		1	10	
				
Time from first positive swab				
Mean (SD)		5.16 (6.28)	11.67 (6.76)	<0.001
Median (Q1, Q3)		0.00 (0.00, 10.00)	10.00 (9.00, 15.00)	
Range		0.00–33.00	0.00–32.00	
				
Antigen detected (pg/mL)				
Mean (SD)		1,680.20 (2,219.85)	0.57 (0.37)	<0.001
Median (Q1, Q3)		94.45 (5.78, 5,000)	0.55 (0.25, 0.81)	
Range		1.19–5000	0.01−1.84	

a Ag, antigen; SD, standard deviation.

The antigen test from the nasopharyngeal swabs described above yielded positive results for 169 (65.3%) samples, while no antigen was detected in 90 of them (34.7%) (Table 1).

When subjects were divided according to positive or negative results for the antigenic test (Table 1), the clinical status in the antigen-positive subjects seemed to move slightly towards a higher frequency of symptoms. In addition, the *C_T_* (threshold cycle) of the molecular test was significantly (<0.001) lower in the antigen-positive group (Table 1). Conversely, the *C_T_* of the molecular test in the antigen-negative group was significantly higher, probably reflecting the descending final phase of the infection.

At the baseline, considering all subjects (*n* = 259), antigen test values were highly negatively correlated with the *C_T_* values of the molecular targets “O” (rho = 0.85, *P* < 0.0001) and “R” (rho = 0.84, *P* < 0.0001), as expected. Since our subjects represented a group of samples collected both at the first diagnosis and following the initial detection, we wondered whether the time of diagnosis affected the detection of viral antigen. Indeed, the time from the first positive swab was significantly (<0.001) shorter (5.16 ± 6.26 days) in the group of antigen-positive subjects (Table 1) compared to that in the antigen-negative group (11.67 ± 6.76 days). Furthermore, 77 of the 90 antigen-negative samples were collected following initial diagnosis, and showed an even longer time (13.47 ± 5.28 days) from the first positive swab (data not shown).

**FIG 1:**
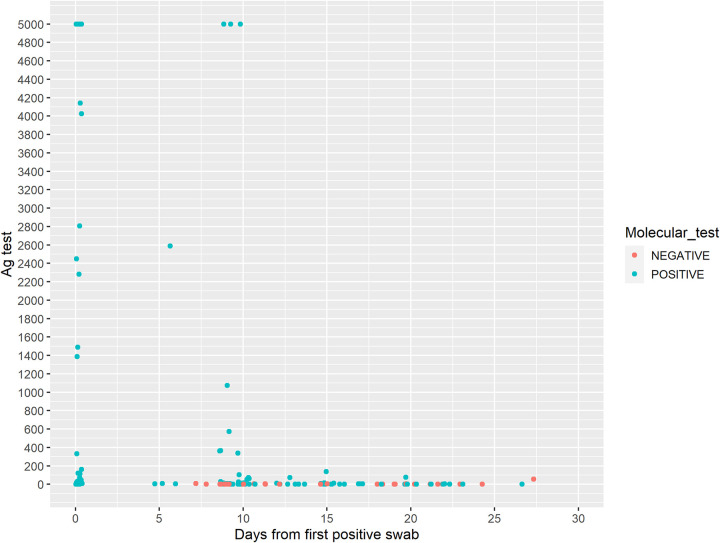
Observations of antigenic (Ag) test results of the 77 subjects who were followed longitudinally, according to their concomitant molecular test results. Antigenic values are expressed in pg/mL. Time is expressed in days from the first positive swab. Different colors indicate different molecular test results (blue, positive; red, negative).

**FIG 2:**
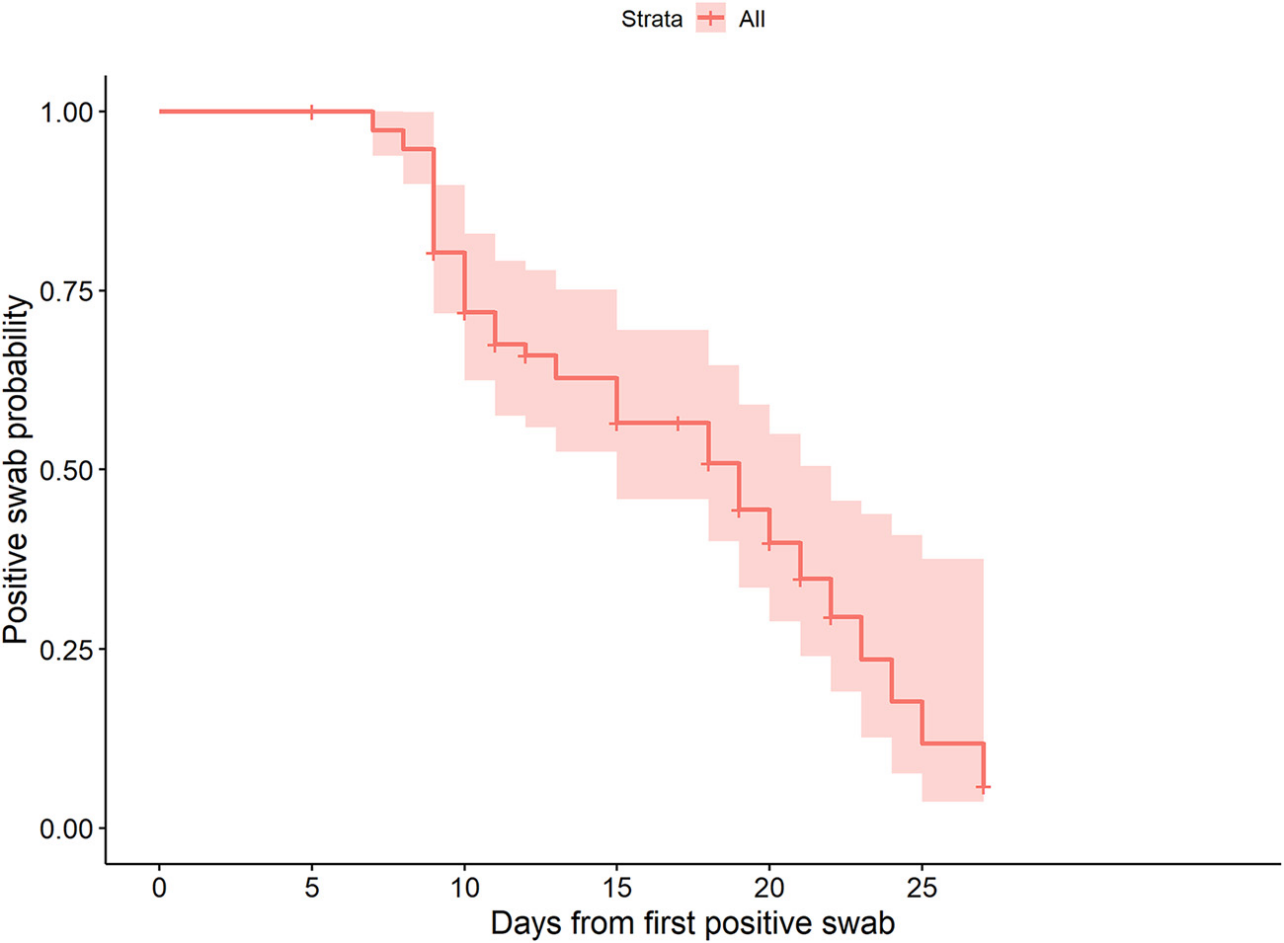
Kaplan-Meier plot showing time to negative swab. Time is expressed in days from first positive swab.

To better understand the viral antigen kinetics in the swab and their meaning, we decided to perform a prospective study. We enrolled 77 newly diagnosed subjects who had positive results for both the molecular and the antigenic tests. These were longitudinally followed-up for a median time of 15 days (range: 5 to 27 days) for a median of 3 visits (range: 2 to 6). At each visit, including the baseline, both molecular and antigenic tests were performed on subjects. It appears from our data that the time of negativization has different kinetics in the two test types, being faster in the antigenic test than in the molecular test: Fig. 1 shows longitudinal observations of antigenic tests according to the concomitant molecular test results. Longitudinal follow-up shows how the negativization of the molecular test is a complicated and unpredictable process, with weak positive results persisting for a long time, with a median positive swab time of 19 days (95% confidence interval (CI):15 to 22 days, Fig. 2).

Antigenic test result values, considered as a time-dependent covariate and log-transformed, were highly associated with the time to negative swab with a hazard ratio (HR) of 0.56 (95% CI: 0.47 to 0.68, *P* < 0.0001) and with good prediction ability (concordance index [C-index]: 0.87); each increment of 1 log-unit in antigenic test values reduced the probability of a negative swab by 44%.

**FIG 3:**
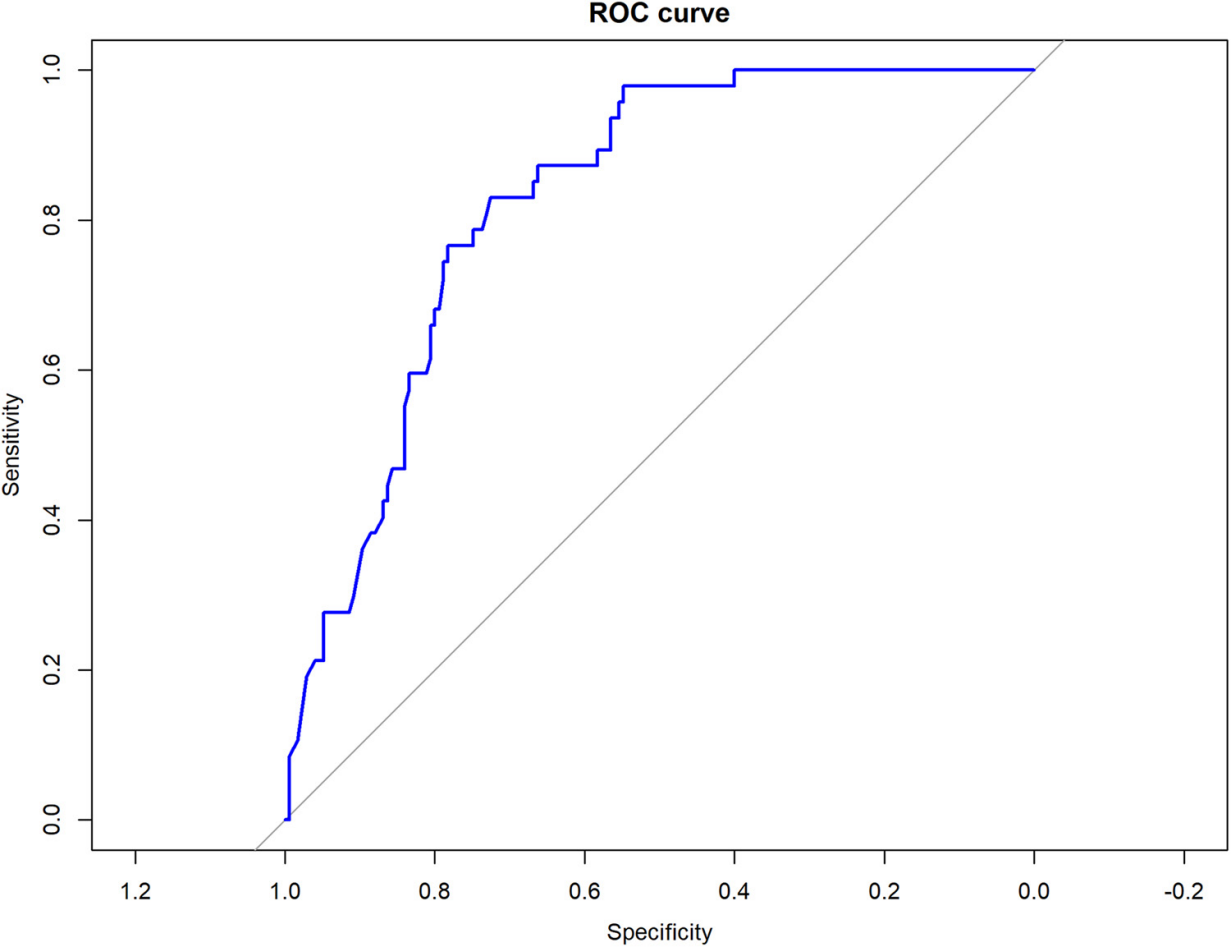
Receiver operating characteristic (ROC) curve analysis. Diagnostic measures, i.e., sensitivity and specificity, are reported.

Regardless of time, a ROC curve analysis showed that the antigenic test had a very good discrimination ability to classify negative swabs, with an area under the ROC curve (AUC) of 0.83 (Fig. 3). The optimal cutoff which jointly maximized sensitivity and specificity was 1.55, resulting in an overall accuracy of 0.75, a sensitivity of 0.73, a specificity of 0.83, a negative predicted value (NPV) of 0.45, and a positive predicted value (PPV) of 0.94.

After dichotomizing the antigenic test according to the previously determined cutoff value of 1.55, the time-dependent covariate Cox model again suggested a highly significant association between antigenic test values and time to negative swab for the molecular test: a subject with an antigenic test value lower than 1.55 had almost a 13-fold greater probability to also have a negative result for the molecular test compared to a subject with an antigenic test value higher than 1.55, as shown by an HR of 12.97 (95% CI: 5.31 to 31.71, *P* < 0.0001), while preserving a good discrimination ability (C-index: 0.80).

## DISCUSSION

An accurate SARS-CoV-2 molecular diagnostic, tightly linked to tracing subjects in contact with infected people, has greatly helped to limit the spread of the COVID-19 pandemic. The high sensitivity of molecular methods greatly enhanced the possibility of detecting even a few copies of the viral genome. This is particularly important in detecting the onset of infection.

However, the wide use of molecular tools poses important and yet unresolved questions. So far, the nature of the weak positive signals which may persist for weeks is unclear. If, on the one hand, it is impossible to rule out the possibility of false positives or, on the other hand, the weak positivity to molecular targets, or to some of them, this does not necessarily mean that complete viral genomes are still present. This is not a trivial issue since it is tightly linked to the possible infectivity of positive subjects. A decline in infectivity beyond 8 to 10 days from the onset of illness or the first positive RT-PCR result in asymptomatic subjects (7–8) has been clearly described in experiments in which viruses from clinical samples were propagated on cultured cells. Although this type of tool represents a proxy for clinically significant infectivity, it is nevertheless suggestive of its decrease during the couple of weeks after the infection peak.

Unfortunately, it is difficult to reconcile the latter observations with the long-term persistence of weak positive results for the molecular test, which has been reported in several subjects; therefore, the moment at which infectivity declines to a negligible level still remains unclear.

This lack of evidence has prompted the Italian Ministry of Health (11) to state that people who, although no longer presenting symptoms, continue to test positive for the molecular test for SARS-CoV-2 but have an absence of symptoms for at least 1 week, will be able to stop isolation 21 days after the onset of symptoms. The difficulty in monitoring the effective kinetics of infection demands new efficient and reliable SARS-CoV-2 testing methods which take into account not only molecular sensitivity but also the ability to pinpoint the different phases of the clinical status of the subject.

The Lumipulse SARS-CoV-2 antigen assay, based on chemiluminescence enzyme immunoassay (CLEIA) technology and able to detect and quantitatively estimate the presence of SARS-CoV-2 nucleocapsid protein (NP) might respond to some of these issues. It is a fast, easy to handle, and inexpensive test that may be very useful in settings where a quick response is required. Since it is not a point-of-care-designed assay, it must be utilized in clinical laboratories. However, due to its slightly lower sensitivity compared to the molecular assay, i.e., 92%, as declared by the manufacturer, it is preferably intended for accurate screening, when a reliable result is critical; for example, for surveillance of health care workers or admittance of subjects to the emergency department in the absence of clear COVID-19 symptoms. In this work, we have explored the possibility of also using the Lumipulse SARS-CoV-2 antigen assay in diagnosis and follow-up with infected subjects.

Antigen-positive subjects seem to comprise, at least in our population, a more consistent portion of symptomatic subjects. The test appears positive in the early phases of infection, making it suitable for diagnostic and screening use. The latter feature is similar to molecular testing. However, the negativization kinetic renders the antigenic test quite different from the molecular test. This is a rather fast and uniform process and, in the prospective analysis, seems not to be affected by the varying positivity observed in the molecular test. The longitudinal observation of antigenic test levels was proven, in our study, to be highly and accurately (C-index = 0.87) predictive of future negative molecular test results, suggesting greater sensitivity in capturing the negativization event and maybe the loss of infectivity early. The suggested cutoff for the antigenic test is, of course, data-driven and needs to be validated in the future and in larger studies, but our study suggests its potential promising clinical usefulness.

The data we have shown in this work, although preliminary, pinpoint the Lumipulse SARS-CoV-2 CLEIA antigen assay as a possible optimal, accurate, and fast tool for follow-up with SARS-CoV-2-positive subjects. We might speculate that the negativization pattern of this test is very similar to that of the clinical loss of infectivity. However, although this is a fascinating hypothesis, more data will be required to definitely prove it. The latter hypothesis is only one of some issues and shadow areas that a more extended study may help to enlighten; such as, for example, the nature of subjects who have molecular test positive results but retain negative antigen test results, despite being in the early phase of infection.

## MATERIALS AND METHODS

### Patients and samples.

Nasopharyngeal swabs were collected at patients’ homes by the Public Hygiene and Prevention Service (SISP) of the province of Lecce (Puglia region, Italy) and sent for analysis to the Molecular Virology Laboratory of Tricase Hospital (Pia Fondazione di Culto e Religione Cardinale Giovanni Panico).

All samples were collected exclusively using nylon-flocked swabs in viral transport medium (UTM kit, Copan, Italy, S.p.A.) and sent to the laboratory, stored at 4°C, in accordance with national guidelines (Istituto Superiore di Sanità, Italian National Institute of Health). All samples were processed within 3 h of swab collection.

In addition, 259 positive samples identified by RT-PCR from 25 January to 18 February 2021 were subjected to antigenic testing. In addition, 77 subjects were longitudinally followed-up between 25 January and 11 March 2021 until clinical recovery based on negative molecular test results; these were subjected to antigenic tests as well.

This study was conducted in accordance with ethical standards and with the Declaration of Helsinki and its later amendments. Sample information, together with the data of subjects for whom SARS-CoV-2 testing was performed (i.e., age and sex), were recorded in an anonymous database by changing sensitive data into alpha-numeric codes.

### Viral RNA extraction and amplification.

Total viral RNA was automatically extracted and amplified through the InGenius (ELITechGroup S.p.A., Torino, Italy, INT030) sample-to-result platform, which integrates extraction, amplification, and result interpretation, allowing a turn-around time of 3 h and reduced hands-on time.

In accordance with the manufacturer’s instructions, the reaction mix was manually prepared using a SARS-CoV-2 ELITe MGB kit (RTS170ING; ELITechGroup S.p.A., Torino, Italy) and loaded onto the system with the reagents of the other kit.

The RNA was extracted by InGenius from 200 μL of viral transport medium. During automatic extraction, the nucleic acid released from the lysed cells binds specifically and exclusively to magnetic beads, and contaminants are removed in subsequent washes. Finally, the viral RNA was eluted in 100 μL elution buffer and immediately subjected to one-step RT-PCR for molecular detection of SARS-CoV-2.

This assay, with a double-target design, is able to identify the RNA of two specific genomic regions of SARS-CoV-2: the *RdRp* and *ORF8* genes. These genes are detected by specific probes using TaqMan MGB technology labeled with different fluorophores. The RdRp probe, stabilized by the MGB group, is labeled with AP593 fluorophore, while the ORF8 probe is labeled with FAM fluorophore (MGB), and both probes are quenched by a non-fluorescent moiety.

In addition, the SARS-CoV-2 ELITe MGB kit allows amplification and detection of the cellularity, extraction, and inhibition control based on the human RNase P gene as an endogenous internal control, using a specific probe labeled with AP525 fluorophore (MGB).

The amplification program includes the following phases: denaturation (95°C for 10 s), annealing (60°C for 30 s), extension (72°C for 20 s) repeated for 45 cycles, preceded by reverse transcription phase at 45°C for 20 min and an initial denaturation at 95°C for 2 min.

At the end of each reaction, assay results were automatically interpreted by the system, as established by the ELITe InGenius Software algorithm and the assay protocol parameters.

The sensitivity and specificity of this kit are both 100%, as declared by the manufacturer.

### SARS-CoV-2 antigen test.

Antigen testing of nasopharyngeal swabs was performed using the Lumipulse SARS-CoV-2 Ag kit (Fujirebio Inc., Tokyo, Japan) on the Lumipulse G600 II automated immunoassay analyzer (Fujirebio).

The Lumipulse SARS-CoV-2 Ag system uses monoclonal antibodies directed against the SARS-CoV-2 protein N, and is designed to perform double-antibody immunodiagnostic tests (Sandwich assay) for the detection and quantitative determination of SARS-CoV-2 antigen in samples taken from the respiratory tract using the CLEIA method.

We directly used 500 μL of viral transport medium from each nasopharyngeal swab, previously centrifuged at 2,000 × *g* for 5 min, to measure antigen levels on the Lumipulse G600 II automated analyzer. The kit’s disposable immunoreaction cartridges, containing the sample treatment solution, antibody-coated magnetic particle solution, and enzyme-labeled antibody solution, were automatically transferred to the immunoreaction unit. The treatment solution and the sample were consecutively aspirated using a single tip. The mix was added to the solution of particles coated with anti-SARS-CoV-2 murine monoclonal antibodies with the formation of Ag-Ab immune complexes.

After an initial washing step, the enzyme conjugate was dispensed into the immunoreaction unit containing a solution of anti-SARS-CoV-2 murine monoclonal antibodies labeled with alkaline phosphatase (ALP), which binds to the N antigen of the particles to form additional immune complexes. Following a second wash step, a substrate solution containing 3-(4’-methoxyspiro[adamantane-2,3’-[1,2]dioxetan]-4’-yl)phenyl dihydrogen phosphate) (AMPPD) was added. The last step takes place in the photodetection unit, where the photons emitted by the specific luminescence reaction (at a maximum wavelength of 477 nm) are measured in proportion to the amount of viral antigen present in the sample.

According to the manufacturer’s information, the cutoff value for the Lumipulse G SARS-CoV-2 Ag, the limit of detection (LOD), and the limit of quantification (LOQ) considered in this study are 1.34 pg/mL, 0.19 pg/mL, and 0.60 pg/mL, respectively.

### Statistical methods.

Baseline clinical and demographical characteristics of the subjects were reported as means and standard deviations (or median and range) and frequency and percentage of continuous and categorical variables, respectively. Group comparisons were carried out using chi-square and unpaired *t* tests for categorical and continuous variables, respectively.

Correlations between baseline antigen test values and *C_T_* (threshold cycle) values “O” and “R” were estimated using Spearman’s correlation coefficients.

The time to negative swab was analyzed using the Kaplan-Meier method for graphical purposes and the proportional hazards Cox model to assess its association with time-dependent antigenic test results. Ag test values were log-transformed before Cox analyses. Risks were reported as hazard ratios along with their 95% CI. The discrimination ability of the antigenic test was measured by the C-index for survival data.

Receiver operating characteristic curve analysis was performed to assess the diagnostic accuracy of the antigenic test in classifying negative and positive swabs. The area under the ROC curve was computed. The optimal cutoff which jointly maximized specificity and sensitivity was estimated. We reported diagnostic measures, i.e., sensitivity, specificity, negative predicted value, and positive predicted value, at the optimal cutoff, defined as described above.

The time-dependent covariate Cox analyses were repeated using the dichotomized version of the antigenic test, according to the optimal cutoff, in order to give a clear and direct clinical interpretation.

A *P* value of <0.05 was considered statistically significant. All analyses were performed using R (version 4.0.2).
